# The impact of serum concentration‐guided digoxin therapy on mortality of heart failure patients: A long‐term follow‐up, propensity‐matched cohort study

**DOI:** 10.1002/clc.23500

**Published:** 2020-11-03

**Authors:** Balázs Muk, Máté Vámos, Péter Bógyi, Barna Szabó, Miklós Dékány, Dénes Vágány, Zsuzsanna Majoros, Tünde Borsányi, Gábor Zoltán Duray, Róbert Gábor Kiss, Noémi Nyolczas

**Affiliations:** ^1^ Dep. of Cardiology Medical Centre Hungarian Defence Forces Budapest Hungary; ^2^ Department of Medicine and Cardiology Center University of Szeged Szeged Hungary; ^3^ Heart‐Lung‐Physiology Clinic Örebro University Hospital Örebro Sweden; ^4^ Doctoral School of Clinical Medicine University of Szeged Szeged Hungary

**Keywords:** digoxin, drug therapy, heart failure, mortality, serum digoxin concentration

## Abstract

**Background:**

Recently published studies suggested that digoxin may increase mortality in heart failure with reduced ejection fraction (HFrEF). However, in the vast majority of former trials serum digoxin concentration (SDC) was not measured and therapy was not SDC‐guided.

**Aim:**

To assess the impact of SDC‐guided digoxin therapy on mortality in HFrEF patients.

**Methods:**

Data of 580 HFrEF patients were retrospectively analyzed. In patients on digoxin, SDC was measured every 3 months and digoxin dosage was SDC‐guided (target SDC: 0.5‐0.9 ng/mL). All‐cause mortality of digoxin users and nonusers was compared after propensity score matching (PSM).

**Results:**

After 7.1 ± 4.7 years follow‐up period (FUP) all‐cause mortality of digoxin users (n = 180) was significantly higher than nonusers (n = 297) (propensity‐adjusted HR = 1.430; 95% CI = 1.134‐1.804; *P* = .003). Patients having SDC of 0.9 to 1.1 ng/mL (n = 60) or > 1.1 ng/mL (n = 44) at any time during the FUP had an increased risk of all‐cause mortality (HR = 1.750; 95% CI = 1.257‐2.436, *P* = .001 and HR = 1.687; 95% CI = 1.153‐2.466, *P* = .007), while patients having a maximal SDC < 0.9 ng/mL (n = 76) had similar mortality risk (HR = 1.139; 95% CI = 0.827‐1.570, *P* = .426), compared to digoxin nonusers.

**Conclusions:**

According to our propensity‐matched analysis, SDC‐guided digoxin therapy was associated with increased all‐cause mortality in optimally treated HFrEF patients, especially with SDC ≥0.9 ng/mL. These results reinforce the expert opinion that digoxin in HFrEF can only be used among carefully selected patients with close SDC monitoring.

## 1 BACKGROUND

Digoxin is one of the oldest drugs in the cardiology armamentarium. The main indication for its application is heart failure (HF) and atrial fibrillation (AF). Despite the extensive use of digoxin, only one randomized placebo‐controlled clinical trial (RCT) has examined the effect of digoxin on morbidity and mortality.^1^ In the DIG (Digitalis Investigation Group) trial, the use of digoxin did not modify all‐cause mortality, although a significant reduction in hospitalization due to worsening HF was observed in HF patients with sinus rhythm (SR). Of note, there has been no RCT assessing mortality and morbidity in patients with AF.

Since the DIG trial observational studies,^2^, ^3^, ^4^, ^5^ post‐hoc analyses of RCTs,^6^, ^7^, ^8^, ^9^ and meta‐analyses^10^, ^11^, ^12^ have been published with the goal of analyzing the mortality effect of digoxin in HF and/or AF. Most of these nonrandomized publications indicated a potential increase in mortality associated with digoxin. However, in most of these studies serum digoxin concentration (SDC) was not measured. The most recent meta‐analysis including more than 825 000 patients, confirmed that digoxin use is associated with increased mortality in patients with AF and HF.^13^ Notably, only 10 of the 37 studies reported data on daily digoxin dose and/or data on SDC.^1^, ^3^, ^14^, ^15^, ^16^, ^17^, ^18^, ^19^, ^20^, ^21^

In the aforementioned publications remains the concern that the mortality‐increasing effect of digoxin may be related to the lack of the control of SDC and consequently elevated SDCs. Furthermore, due to the potentially incomplete adjustment of all the potentially influencing confounders the observed digoxin‐associated mortality increase might be due to the more frequent use of this drug in sicker patients.^22^

According to the recent guidelines for HF,^23^ the use of digoxin has decreased significantly, however digoxin still affects large patient populations.^24^

Given the lack of studies that have evaluated the effect of SDC‐guided digoxin therapy, we aimed to assess the effect of digoxin on long‐term survival in a HF with reduced ejection fraction (HFrEF) patient population, where digoxin dose was regularly adjusted based on SDC.

## 2 METHODS

### 2.1 Patient population

Data from consecutive HFrEF patients managed at the HF outpatient clinic (HFOC) of the Medical Center of Hungarian Defense Forces between 1 January 2007 and 31 December 2017 were collected retrospectively. Demographic data and clinical information were gathered from outpatient records.

Patients were considered to suffer from HFrEF if the left ventricular ejection fraction (LVEF) was <40%. LVEF was measured by echocardiography using the biplane Simpson method. Patients were classified as digoxin users if digoxin was administered at the time of the initiation of HFOC care and digoxin therapy was applied without interruption during the follow‐up period (FUP). Patients who received digoxin at the time of referrals, but digoxin therapy was discontinued afterward during the FUP were excluded from the study. Patients were considered to be new digoxin users if digoxin was initiated at the first visit at the HFOC. Patients who did not receive digoxin at baseline, but digoxin treatment was introduced during the FUP were excluded from the study. Patients were considered to be nondigoxin users if digoxin was not used and not started at baseline. Digoxin initial dosing was calculated with a standardized method.^25^ Afterwards SDC was measured every 3 months and the dose was adjusted according to it. The goal therapeutic range of SDC was 0.5 to 0.9 ng/mL.^26^ SDC samples were usually taken after 4 to 6 hours of oral administration. During follow‐up we made every effort to apply guideline‐recommended therapy to every patient.

The study was approved by the institutional review board and complies with the ethical guidelines of the Declaration of Helsinki.

### 2.2 Study end points

The outcome measure of this study was time to all‐cause mortality. This parameter was compared between digoxin users and nonusers across the whole patient population and after propensity score matching (PSM). Digoxin users were also divided into three groups based on the maximal SDC measured during follow‐up (maxSDC < 0.9 ng/mL, 0.9 ≤ maxSDC < 1.1 ng/mL, maxSDC ≥ 1.1 ng/mL) and survival was compared among these subgroups of the propensity‐adjusted population. Furthermore, the effect of SDC‐guided digoxin therapy on all‐cause mortality was assessed in new digoxin users and in patients with AF and SR also in the propensity‐adjusted population. Mortality data were obtained from the database of the National Health Insurance Fund of Hungary.

### 2.3 Statistical analysis

Statistical analysis was performed using SPSS Statistics software, Version 23.0 (IBM, Armonk, NY) with the R software plug‐in (The R Foundation, Version 3.1.0) for PSM.

Continuous variables were expressed as mean ± SDs, and differences were compared using 2‐sample *t*‐tests or the Mann‐Whitney *U* test, as appropriate. Categorical variables were expressed as counts and percentages and differences were assessed with the chi square test.

To assess the effects of SDC‐guided digoxin on survival, the Cox proportional hazards regression model was used. The variables included in the multivariate regression analysis are the best‐known parameters influencing prognosis in HFrEF. The statistical models were adjusted for potential baseline confounders, including sex, age, etiology of HFrEF, AF, hypertension, diabetes mellitus, New York Heart Association functional class, LVEF, QRS width, heart rate, serum creatinine level, hemoglobin level, beta‐blocker (βB), angiotensin‐converting enzyme inhibitors (ACEi)/angiotensin receptor blockers (ARB), mineralocorticoid receptor antagonists (MRA), amiodarone, device use. Mortality risk assessment was also repeated among propensity‐score‐matched patient groups. Patients receiving digoxin were matched in a 1:2 ratio with patients not treated with digoxin using the nearest neighbor matching method with a caliper of 0.2 by applying the baseline characteristics listed above for the multivariate Cox regression. We also assessed the digoxin‐associated mortality risk among the following subgroups of the propensity‐score adjusted patient cohort: the subgroups defined by maximal SDC measured during follow‐up (maxSDC <0.9 ng/mL, 0.9 ≤ maxSDC <1.1 ng/mL, maxSDC ≥ 1.1 ng/mL), patients with SR or AF at baseline, and patients with newly prescribed digoxin at baseline visit.

Survival curves were constructed according to the Kaplan‐Meier method and compared with the Cox proportional hazard model and the Wald test for the multivariate analyses. Two‐sided *P* values of <.05 were considered statistically significant.

## 3 RESULTS

### 3.1 Patients characteristics

The baseline characteristics of the patients of the total cohort and patients after PSM with and without digoxin treatment are presented in Table 1. From the total cohort, 185 patients received digoxin at the time of first visit to the HFOC. As expected, digoxin users suffered more often from AF than nondigoxin users (41.1% vs 21.3%) and had more decreased ejection fraction (26.4 ± 6.5% vs 28.0 ± 6.6%). Ischemic etiology was more frequent among nondigoxin users. There was also a significant difference between the two groups regarding baseline device use: significantly more digoxin‐treated patients had a previously implanted ICD or CRT‐P/D compared to nonusers (13.0% vs 7.6%). Regarding drug treatment applied at baseline, the minority of patients received guideline‐recommended therapy of HFrEF. The majority of evaluated patients were referred to our HFOC from secondary care physicians and general practitioners. Therefore, many of them were treatment naïve or undertreated at the time of referrals. In 40.2% of patients a βB, in 40.3% an ACEi/ARB, and in 36.7% an MRA was applied. After the treatment optimization period of 3 to 6 months, the proportion of patients receiving the neurohormonal antagonists increased significantly. In the total cohort the utilization of βB and ACEi/ARB was also 88.4%, while MRA was applied in 57.6%. It has to be highlighted that, the proportion of patients receiving target doses of these mortality‐reducing agents also increased remarkably (46.7% of βB‐treated and 41.5% of ACEi/ARB‐treated patients), which results were significantly favorable than observed in the recently published registry data.^24^ The mean daily digoxin dose during follow‐up was 111 ± 50 μg. During the period of the study, the angiotensin receptor‐neprilysin inhibitor treatment was still not available.

**TABLE 1:** Baseline characteristics before and after propensity score matching for patients with and without digoxin therapy

	Before propensity score matching (580)				After propensity score matching (477)			
		Pts without digoxin (395)	Pts with digoxin (185)	*P*‐value		Pts without digoxin (297)	Pts with digoxin (180)	*P*‐value
Male	443 (76.4%)	300 (75.9%)	143 (77.3%)	.722	363 (76.1%)	224 (75.4%)	139 (77.2%)	.655
Age (mean ± SD)	61.2 ± 13.0	61.6 ± 13.1	60.2 ± 12.6	.201	60.7 ± 13.2	60.8 ± 13.6	60.5 ± 12.7	.693
Ischemic etiology	272 (46.9%)	198 (50.1%)	74 (40.0%)	.023	199 (41.7%)	128 (43.1%)	71 (39.4%)	.433
Atrial fibrillation	160 (27.6%)	84 (21.3%)	76 (41.1%)	<.001	154 (32.3%)	83 (27.9%)	71 (39.4%)	.009
Hypertension	420 (72.4%)	294 (74.4%)	126 (68.1%)	.112	332 (69.6%)	209 (70.4%)	123 (68.3%)	.639
Diabetes mellitus	203 (35.0%)	141 (35.7%)	62 (33.5%)	.607	170 (35.6%)	109 (36.7%)	61 (33.9%)	.534
NYHA (mean ± SD)	3.1 ± 0.8	3.1 ± 0.8	3.2 ± 0.7	.613	3.1 ± 0.8	3.1 ± 0.8	3.1 ± 0.7	.714
LVEF (%) (mean ± SD)	27.5 ± 6.6	28.0 ± 6.6	26.4 ± 6.5	.003	26.8 ± 6.6	27.0 ± 6.7	26.6 ± 6.4	.384
QRS width (ms) (mean ± SD)	124 ± 38	122 ± 37	129 ± 39	.063	127 ± 38	127 ± 38	127 ± 38	.974
HR(min^−1^) (mean ± SD)	86.3 ± 19.6	85.1 ± 19.2	89.0 ± 20.0	.026	88.0 ± 20.2	87.3 ± 20.3	89.0 ± 20.1	.375
Creatinine (μmol/l) (mean ± SD)	114 ± 48	113 ± 45	117 ± 53	.177	116 ± 50	116 ± 48	117 ± 53	.713
Hgb (g/L) (mean ± SD)^a^	142 ± 17	141 ± 16	143 ± 17	.116	142 ± 16	141 ± 15	143 ± 17	.153
ß‐blocker	233 (40.2%)	156 (39.5%)	77 (41.6%)	.626	189 (39.6%)	115 (38.7%)	74 (41.1%)	.605
ACEi/ARB	234 (40.3%)	157 (39.7%)	77 (41.6%)	.668	190 (39.8%)	116 (39.0%)	74 (41.1%)	.657
MRA	213 (36.7%)	141 (35.7%)	72 (38.9%)	.453	173 (36.3%)	104 (35.0%)	69 (38.3%)	.465
Amiodarone	44 (7.6%)	27 (6.8%)	17 (9.2%)	.318	42 (8.8%)	25 (8.4%)	17 (9.4%)	.701
CRT/ICD	54 (9.3%)	30 (7.6%)	24 (13.0%)	.038	51 (10.7%)	31 (10.4%)	20 (11.1%)	.818

Abbreviations: ACEi, angiotensin‐converting enzyme inhibitor; ARB, angiotensin receptor blocker; CRT, cardiac resynchronization therapy; Hgb, hemoglobin; HR, heart rate; ICD, implantable cardioverter‐defibrillator; LVEF, left ventricular ejection fraction; MRA: mineralocorticoid receptor antagonist; NYHA: New York Heart Association functional class.

^a^ Available for 467 pts before and 383 pts after propensity score matching.

**FIGURE 1:**
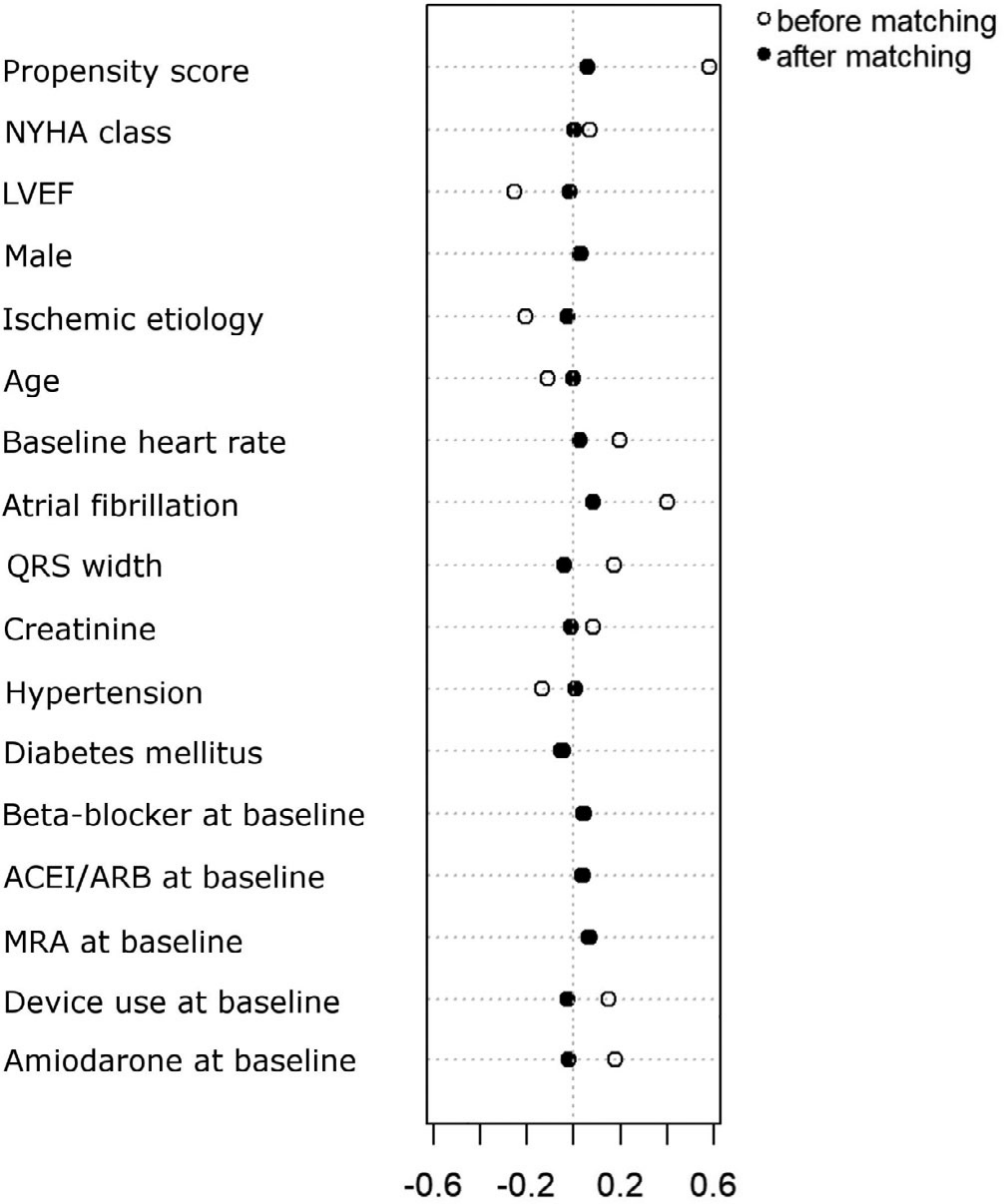
Dotplot of standardized mean differences for 17 baseline characteristics between digoxin users and nonusers, before and after propensity score matching. ACEi, angiotensin‐converting enzyme inhibitor; ARB, angiotensin receptor blocker; LVEF, left ventricular ejection fraction; MRA, mineralocorticoid receptor antagonist, NYHA, New York Heart Association functional class

After applying a 1:2 propensity‐score matching protocol, a cohort of 477 patients was assembled (180 digoxin‐treated and 297 digoxin‐not‐treated patients). Compared with prematched patients, those in the matched cohort were well balanced with respect to the collected baseline risk factors with a standard mean difference less than 20% (Figure 1, Suppl. Figure S1), although patients with digoxin exposure had higher incidence of AF (39.4% vs 27.9%, *P* = .009).

### 3.2 Mortality during follow‐up

During the mean follow‐up of 7.1 ± 4.7 years, from the total cohort 351 patients died, 131 patients out of 185 digoxin users and 220 patients out of the 395 nondigoxin users. The univariate survival analysis of the total cohort showed that digoxin use was associated with an increased risk of all‐cause mortality (HR: 1.453, 95% CI: 1.170‐1.804, *P* = .001). After adjustment for potential confounders in multivariate Cox regression analysis, baseline digoxin use remained an independent predictor of all‐cause mortality (HR: 1.939, 95% CI: 1.512‐2.487, *P* < .001) (Suppl. Table S1).

**FIGURE 2:**
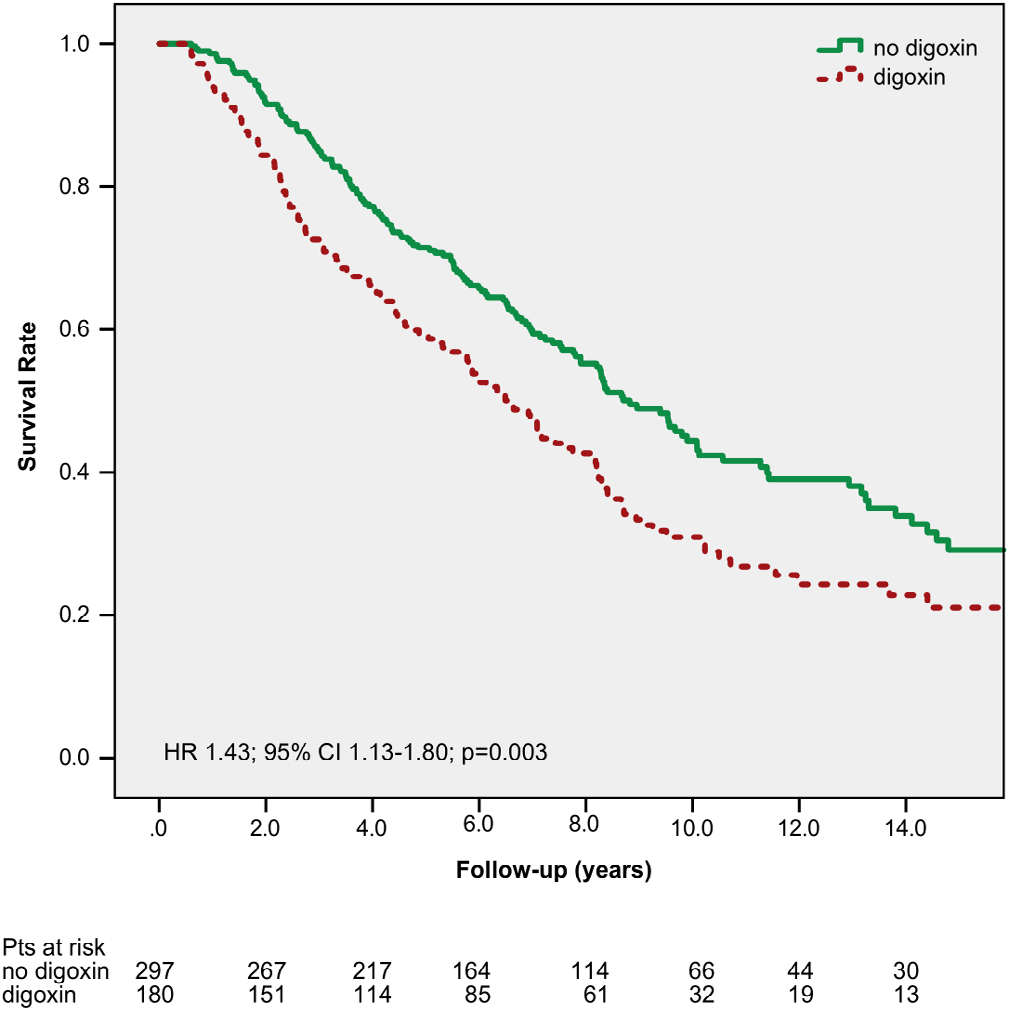
Kaplan–Meier curves for all‐cause mortality by digoxin use (propensity‐matched patients). CI, confidence interval; HR, hazard ratio

In the propensity‐score‐matched patient cohort 126 patients out of the 180 digoxin users and 165 patients out of the 297 nondigoxin users died. The all‐cause mortality of digoxin users was significantly higher than nonusers (propensity‐adjusted HR: 1.430, 95% CI: 1.134‐1.804, *P* = .003) (Figure 2). Beside the baseline digoxin use, sex, age, ischemic etiology, AF, hypertension, diabetes mellitus, NYHA functional class, QRS width, serum creatinine level, amiodarone use, and hemoglobin level were correlated with the survival in the propensity‐score‐matched patient cohort (Table 2).

**TABLE 2:** Predictors of mortality in the propensity‐score adjusted patient cohort (Univariate Cox regression analysis)

	Adjusted HR	CI 95%		*P*‐value
Male	1.736	1.280	2.355	<.001
Age	1.050	1.040	1.061	<.001
Ischemic etiology	2.275	1.798	2.879	<.001
Atrial fibrillation	1.530	1.205	1.942	<.001
Hypertension	1.385	1.070	1.794	.013
Diabetes mellitus	1.423	1.125	1.799	.003
NYHA	1.418	1.215	1.655	<.001
QRS width	1.003	1.001	1.006	.020
Creatinine	1.004	1.003	1.006	<.001
Amiodarone	1.553	1.024	2.357	.038
Hemoglobin	0.984	0.976	0.992	<.001
Digoxin	1.430	1.134	1.804	.003

Abbreviations: CI, confidence interval; HR, hazard ratio; NYHA, New York Heart Association functional class.

### 3.3 Subgroup analyses in the propensity matched population

**FIGURE 3:**
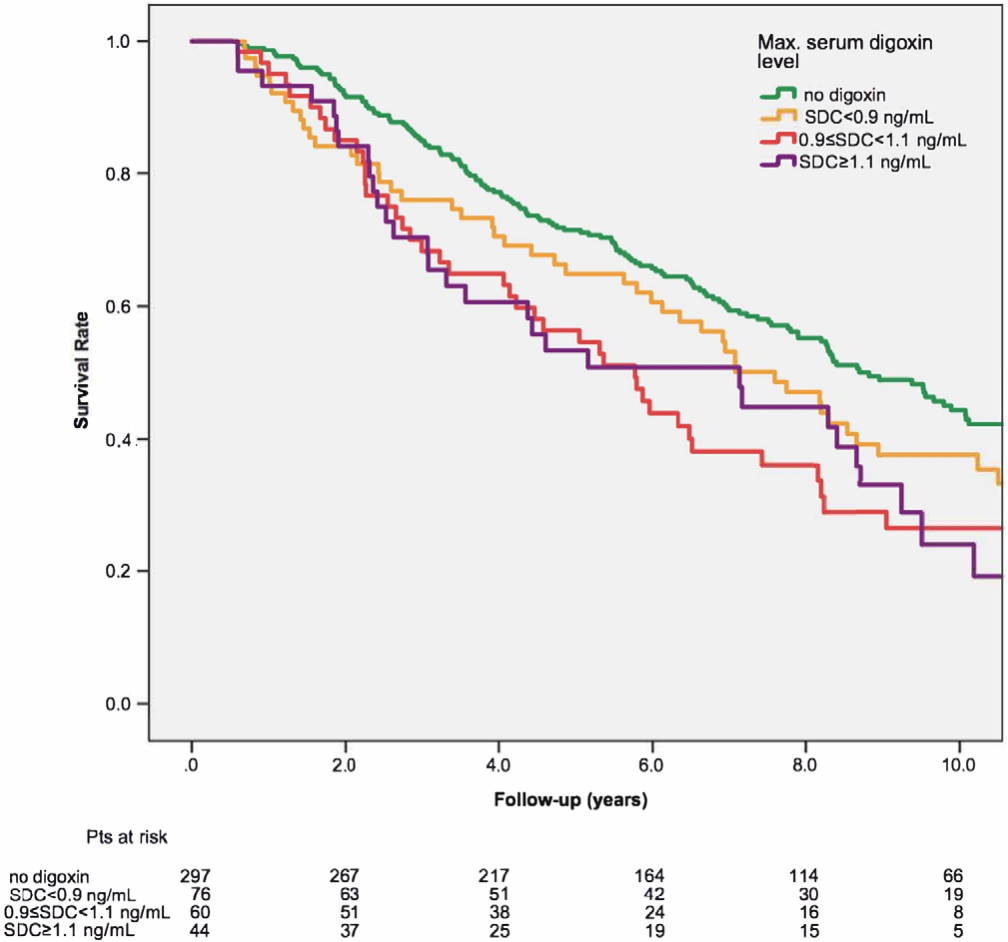
Kaplan–Meier curves for all‐cause mortality by maximal serum digoxin concentration (propensity‐matched patients). SDC, serum digoxin concentration

Those patients who had a maxSDC of between 0.9 and 1.1 ng/mL (n = 60) and patients with maxSDC ≥ 1.1 ng/mL (n = 44) had an increased risk of all‐cause mortality compared to nondigoxin users (HR: 1.750, 95% CI: 1.257‐2.436, *P* = .001 and HR: 1.687, 95% CI: 1.153‐2.466, *P* = .007) (Figure 3). This elevated risk of mortality was not statistically significant in the subgroup of patients with a maxSDC of <0.9 ng/mL (n = 76) (HR: 1.139, 95% CI: 0.827‐1.570, *P* = .426) (Figure 3).

When survival was analyzed according to digoxin use in the subgroup of patients with SR at baseline, we found that digoxin use was associated with an elevated hazard of mortality (propensity‐adjusted HR: 1.553, CI: 1.157‐2.084, *P* = .003) (Suppl. Figure S2a). This phenomenon was not statistically significant among those having AF at baseline (HR: 1.106, CI: 0.756‐1.619, *P* = .604) (Suppl. Figure S2b).

When we analyzed the effect of digoxin among the 123 new digoxin users compared to digoxin nonusers, we found that digoxin resulted in a significant increased risk of all‐cause mortality (HR: 1.371, 95% CI: 1.062‐1.770, *P* = .016) (Suppl. Figure S3).

When digoxin level was analyzed as a continuous variable, SDC was associated with a 14% higher adjusted hazard of death for each 0.5 ng/mL increase (*P* = .0073).

## 4 DISCUSSION

### 4.1 Main findings

In this real‐life, community‐based cohort of optimally treated HFrEF patients, we found that SDC‐guided digoxin therapy was associated with increased all‐cause mortality, especially with SDC ≥ 0.9 ng/mL. In patients with SR and in new digoxin users all‐cause mortality was also significantly elevated compared to patients not treated with digoxin.

### 4.2 Serum‐concentration‐guided digoxin therapy

The narrow therapeutic window for the use of digitalis glycosides is well known. However, most publications that demonstrated an elevated mortality risk associated with digoxin did not report data about daily digoxin dose and/or serum levels. Even in the studies that reported such information, serum digoxin measurements were not performed in a systematic fashion. In the DIG trial SDC was measured only at 4 weeks and 1 year after the start of the study, while digoxin toxicity was followed only by signs and symptoms at 4 months, and every 4 months thereafter.^1^ In a study by Freeman et al. comprising 2891 newly diagnosed HFrEF patients,^17^ SDC was measured at all in 70% of patients and was measured just once in 27% of patients. Consequently, the lack of regular SDC control and/or higher SDC may have contributed to the adverse mortality effect of digoxin observed in these trials.

Our retrospective study demonstrates that even with an extremely close monitoring strategy, which were performed systematically in every patient, it was only possible to maintain SDC below 0.9 ng/mL in 42% of patients during the entire follow‐up. This may be partly due to the pharmacokinetics of digoxin (it eliminates mainly through the kidneys), and the fact that the renal function of HFrEF patients is typically impaired. It therefore appears to be reasonable to use digitoxin instead of digoxin in HFrEF because of its hepatic elimination. Evidence regarding the effects of digitoxin on morbidity and mortality or data about its safe therapeutic range is even more limited. In a single‐center study of 1020 ICD recipients, treatment with digoxin or digitoxin were associated with similarly increased mortality compared to digitalis nonusers.^27^ The ongoing DIGIT‐HF trial will hopefully be able to clarify the place of digitoxin in therapy for HFrEF.^28^ This trial is investigating the hypothesis that digitoxin—at serum concentrations in the lower therapeutic range—reduces mortality and morbidity in patients with HFrEF with or without AF.

### 4.3 Correlation of SDCs and mortality

A post‐hoc analysis of the DIG trial has raised the concern that high SDC (≥1.2 ng/mL) could lead to an increase in all‐cause and cardiovascular mortality, and favorable digoxin effects are only expected in patients with SDC between 0.5 and 0.8 ng/mL.^26^ In the recently published post‐hoc analysis of the ARISTOTLE trial^19^ baseline digoxin use was not associated with an increased risk of mortality compared to patients not treated with digoxin. However, a 56% increase in relative risk of mortality was demonstrated in patients with an SDC ≥ 1.2 ng/mL compared to those who were not on digoxin. The study also found a linear correlation between SDC and all‐cause mortality: an 0.5 ng/mL increase in SDC increased mortality by 19%. This phenomenon was also demonstrated in our analysis, SDC was associated with a 14% higher adjusted hazard of death for each 0.5 ng/mL increase. In contrast to the aforementioned post‐hoc analysis of the ARISTOTLE trial we experienced an increase in mortality risk across the entire patient group before and after PSM. This difference may be explained by the variability in patient populations: in the ARISTOTLE trial every patient had AF, 37.4% of whom suffered from concomitant HF, while in our study every patient had HFrEF and only 27.6% suffered from AF. It may also be noted that in the ARISTOTLE study among patients whose digoxin level was measured at baseline, 76.0% had SDC levels below 0.9 ng/mL, while only 42% of our patient population had maxSCD < 0.9 ng/mL.

In contrast to the DIG study, we could not identify a favorable mortality effect in patients with maxSDC < 0.9 ng/mL. This may be explained by the fact that there were significant differences between our patient population and those cohorts (eg, we included patients with AF also, in contrast to the DIG trial). Moreover, digoxin users had more advanced HF with lower LVEF in our cohort, and the proportion of patients with hypertension or diabetes was higher compared to the DIG trial. It should be also noted that the morbidity‐ and mortality‐reducing drug and device therapies were applied in higher proportion and dose in our patients than they were used in the DIG trial, which also could have modified the possible deleterious effects of digoxin.

### 4.4 Effect of digoxin in patients with AF and SR

The results of studies that evaluated the effect of digoxin on mortality of HFrEF patients in SR and AF are quite controversial. In a meta‐analysis published by Vamos et al, a substantially increased risk of death was associated with digoxin in both HF and AF, although the relative risk of mortality was higher in patients with AF (23% vs 11%).^13^ The post‐hoc analysis of the ARISTOTLE trial also demonstrated a direct correlation between serum digoxin level and overall mortality in patients with AF, which was consistent in patients with HF. However, Hallberg et al—using data from the Registry of Information and Knowledge about Swedish Heart Intensive Care Admissions—did not find a difference in 1 year digoxin‐associated mortality among patients with HF with or without AF. Our study demonstrated increased mortality in digoxin‐treated HFrEF patients in SR but not in patients with AF. The ongoing RATE‐AF trial that is examining the effect of digoxin in AF and HF may clear up this relevant clinical issue.^29^

### 4.5 Effect of digoxin on new digoxin users

Similarly to the post‐hoc analysis of the ARISTOTLE trial^19^ and other previous reports, we also found a significant increase in all‐cause mortality in new digoxin users compared to patients not treated with digoxin (HR: 1.371, 95% CI: 1.062‐1.770). Although this result may be underpowered because of the limited number of new digoxin users, this type of analysis appears to be particularly important, since it reduces the survival bias that is present in most of the observational studies.^13^

### 4.6 Limitations

However, in our nonrandomized patient cohort analysis we aimed to minimize potential confounding factors by carefully adjusting our data along important patient characteristics potentially responsible for worse outcomes using two different statistical methods (ie, adjusted multivariate Cox regression and PSM), residual bias cannot be excluded, as this was pointed by Aguirre Dávila et al in a recently published post‐hoc analysis of the DIG trial.^22^ The observed neutral effect of digoxin in the subgroup of patients with SDC < 0.9 ng/mL on mortality should be interpreted carefully, hence this group represents a small number of patients and has limited statistical power.

The data collection process for our patient cohort started in 2007. Since then, there have been changes in the guideline recommendations regarding the pharmacological and device treatment of HFrEF. These changes may have modified the mortality effect of digoxin.

Our single‐center patient population consisted of only Caucasians. Accordingly, the results of the study do not necessarily apply to patients outside this group.

## 5 CONCLUSIONS

In the current, retrospective, single‐center study, serum‐concentration‐guided digoxin therapy was associated with increased all‐cause mortality in optimally treated HFrEF patients, especially with SDC ≥ 0.9 ng/mL. It can be also concluded that the safe use of digoxin, which does not result in unfavorable outcomes in HFrEF is hardly feasible.

## CONFLICT OF INTEREST

The authors have nothing to disclose.

## Supporting information


**Appendix** S1: Supporting information

## Data Availability

The data that support the findings of this study are available from the corresponding author upon reasonable request.
