# Supplementary material for: The impact of serum concentration‐guided digoxin therapy on mortality of heart failure patients: A long‐term follow‐up, propensity‐matched cohort study
Source: Clin Cardiol. 2020 Nov 3;43(12):1641–8. doi: 10.1002/clc.23500 (PMC7724220; doi:10.1002/clc.23500)

SUPPLEMENTAL MATERIAL

**Supplementary Table 1.**

Legends: Independent baseline predictors of mortality in the total cohort (Multivariate Cox regression analysis)

CI: confidence interval; NYHA: New York Heart Association functional class, HR: hazard ratio.

|  | **adjusted HR** | **95% CI** | | **p-value** |
| --- | --- | --- | --- | --- |
|  |  | **Lower** | **Upper** |  |
| Digoxin | 1.939 | 1.512 | 2.487 | <0.001 |
| NYHA | 1.212 | 1.037 | 1.416 | 0.015 |
| Male | 1.986 | 1.422 | 2.774 | <0.001 |
| Ischemic etiology | 1.738 | 1.338 | 2.257 | <0.001 |
| Age | 1.043 | 1.032 | 1.055 | <0.001 |
| Creatinine | 1.003 | 1.001 | 1.005 | 0.013 |
| Haemoglobin at baseline | 0.985 | 0.977 | 0.993 | <0.001 |

**Supplementary Figure 1.**

Legends:

The absolute standardized differences in mean values of the main clinical baseline parameters before and after propensity score matching
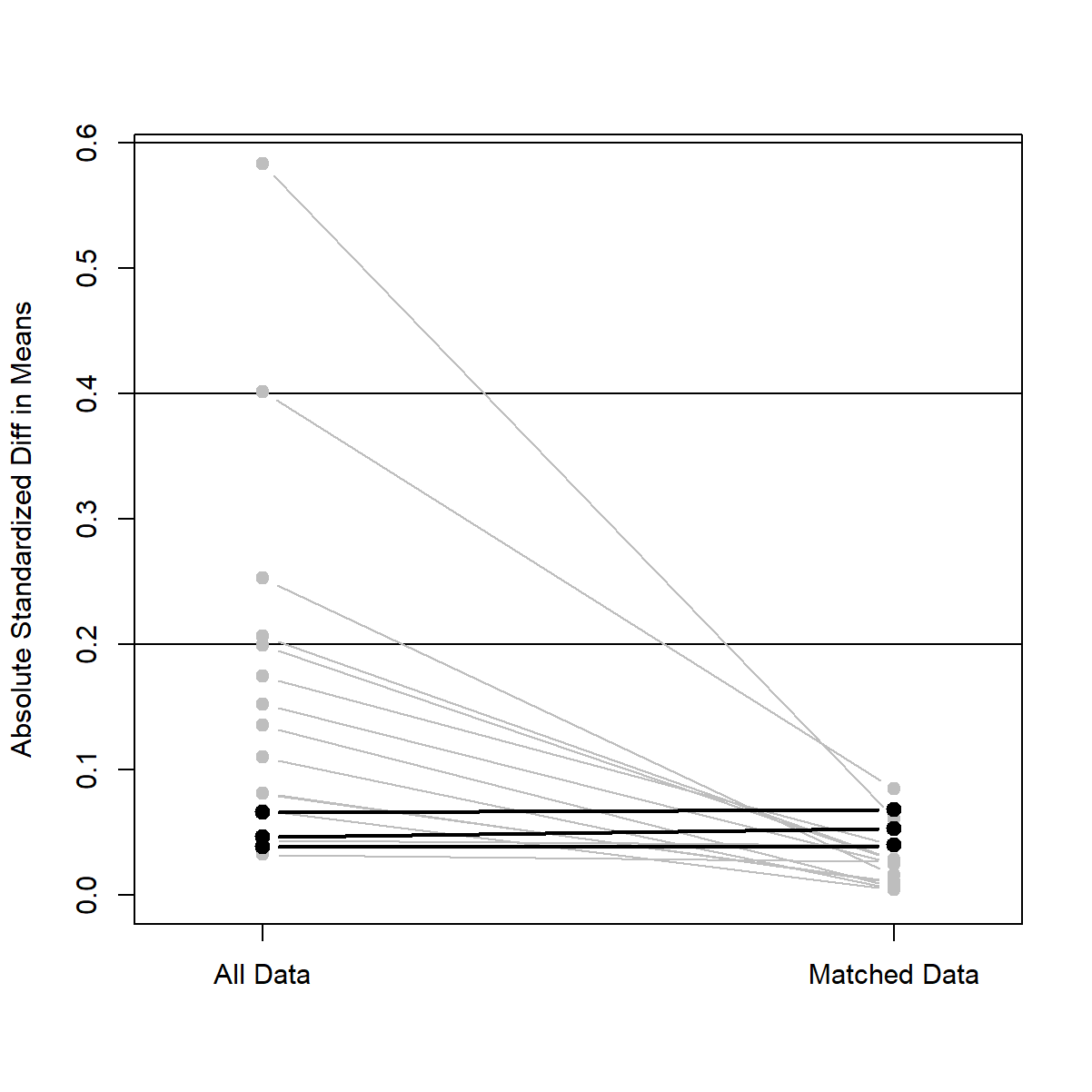


**Supplementary Figure 2a.**

Legends: Kaplan-Meier curves of all-cause mortality by digoxin use in presence of sinus rhythm at baseline (propensity matched patients)

(CI: confidence interval; HR: hazard ratio)

**
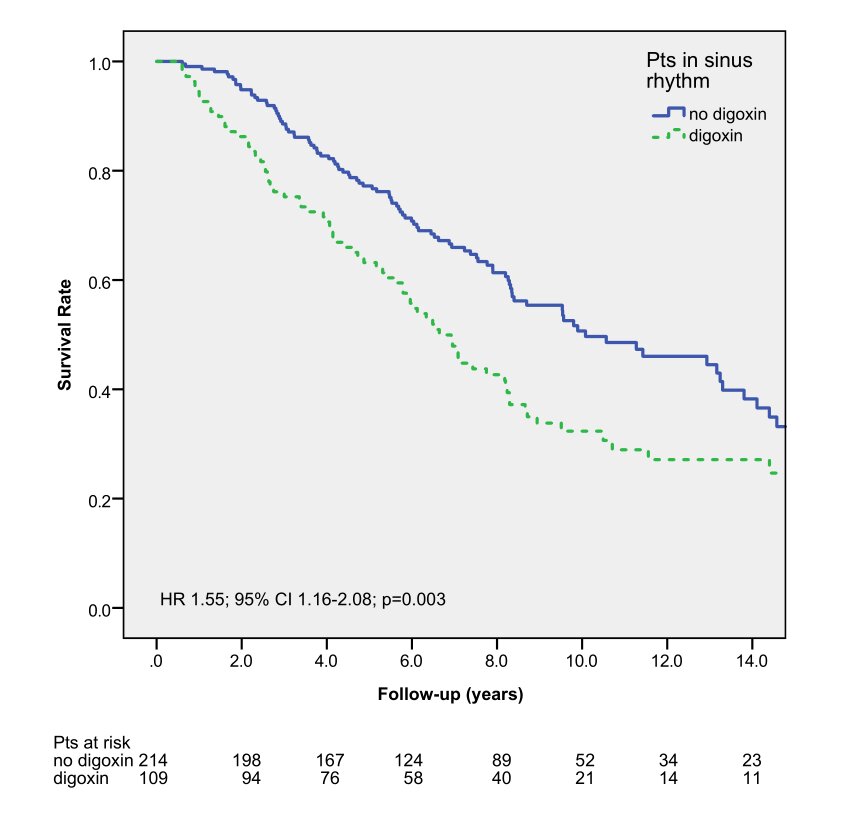
**

**Supplementary Figure 2b.**

Legends: Kaplan-Meier curves of all-cause mortality by digoxin use in presence of atrial fibrillation at baseline (propensity matched patients)

(CI: confidence interval; HR: hazard ratio)


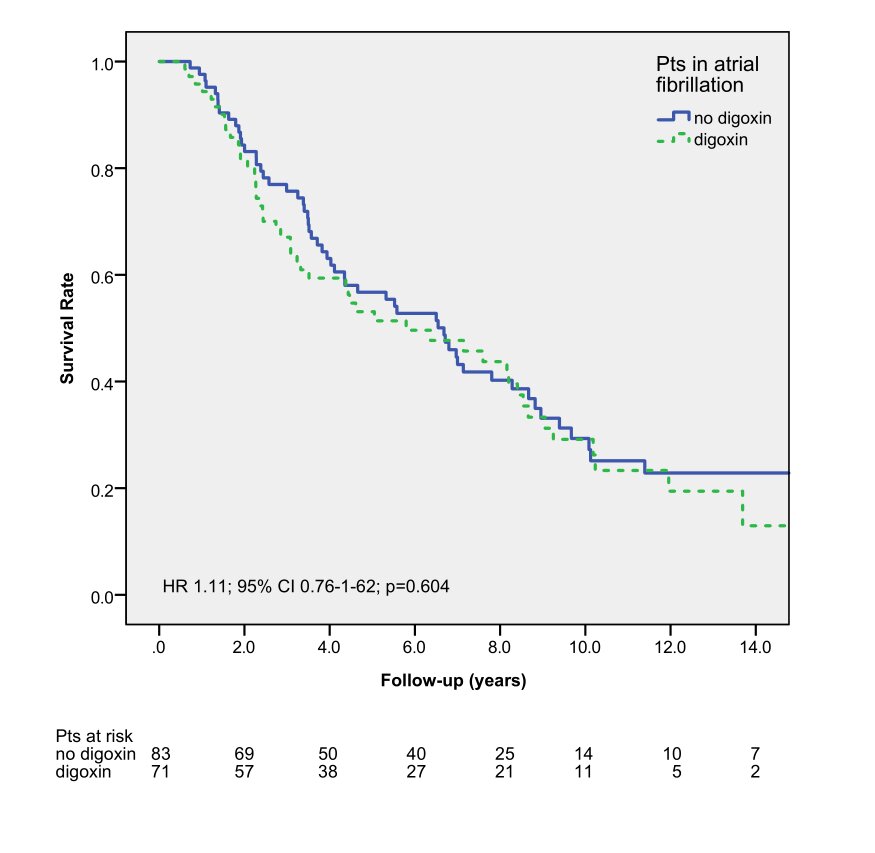


**Supplementary Figure 3.**

Legends: Kaplan-Meier curves for all-cause mortality by digoxin use among new digoxin users (propensity matched patients).

CI: confidence interval; HR: hazard ratio


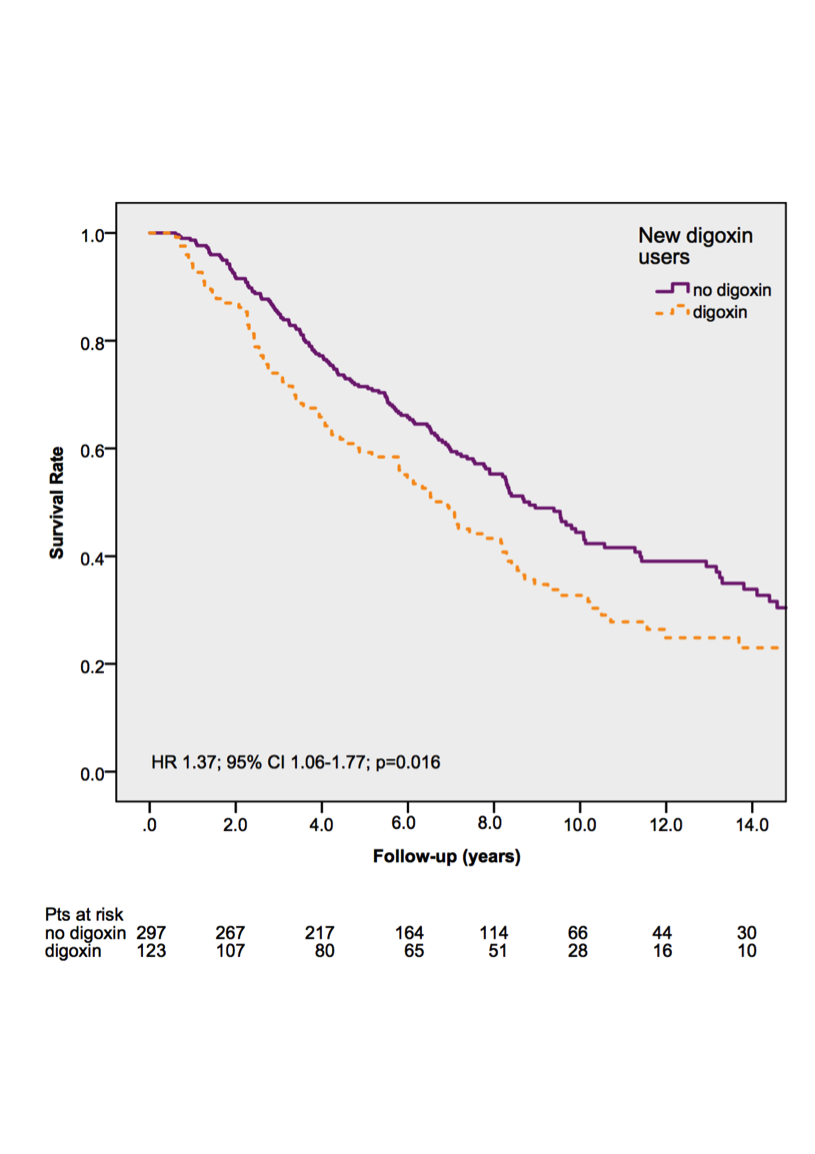

Supplement: Supplementary file 1 — Appendix S1: Supporting information [file CLC-43-1641-s001.docx]
